# Experimental Investigation on the Influence of Fiber Path Curvature on the Mechanical Properties of Composites

**DOI:** 10.3390/ma14102602

**Published:** 2021-05-17

**Authors:** Huaqiao Wang, Jihong Chen, Zhichao Fan, Jun Xiao, Xianfeng Wang

**Affiliations:** 1 School of Mechanical Science & Engineering, Huazhong University of Science and Technology, Wuhan 430074, China; wanghuaqiao666@sina.com (H.W.); 13308656728@189.com (J.C.); 2 College of Materials Science and Technology, Nanjing University of Aeronautics and Astronautics, Nanjing 210016, China; fanzc0422@126.com (Z.F.); nuaaccmexj@126.com (J.X.)

**Keywords:** automated fiber placement, path planning, mechanical properties, experimental study

## Abstract

Automated fiber placement (AFP) has been widely used as an advanced manufacturing technology for large and complex composite parts and the trajectory planning of the laying path is the primary task of AFP technology. Proposed in this paper is an experimental study on the effect of several different path planning placements on the mechanical behavior of laminated materials. The prepreg selected for the experiment was high-strength toughened epoxy resin T300 carbon fiber prepreg UH3033-150. The composite laminates with variable angles were prepared by an eight-tow seven-axis linkage laying machine. After the curing process, the composite laminates were conducted by tensile and bending test separately. The test results show that there exists an optimal planning path among these for which the tensile strength of the laminated specimens decreases slightly by only 3.889%, while the bending strength increases greatly by 16.68%. It can be found that for the specific planning path placement, the bending strength of the composite laminates is significantly improved regardless of the little difference in tensile strength, which shows the importance of path planning and this may be used as a guideline for future AFP process.

## 1. Introduction

In recent years, fiber-reinforced composite materials have been widely used in the automotive industry, aeronautics, astronautics, etc. They have a series of advantages such as high specific strength, good thermal stability, improved corrosion resistance, and well designability [1]. Automated fiber placement (AFP), as an advanced manufacturing technology that has been gradually developed in recent years, has been widely used in the manufacture of various large spacecraft structures, such as satellites and solid rockets, instead of the traditional manual prepreg placement molding. After slitting, the prepreg or the prepreg tape can be independently conveyed and cut by a specific device. A pre-dip tape with a certain width is formed under the pressure of the pressure roller, in which the bandwidth is determined by the number of tows. After heating and softening, under the pressure of the laying head, the prepreg tape is compacted on the mold surface. This technology can greatly improve production efficiency [2,3].

The trajectory planning of the laying path with different angles is the primary task of many new technologies such as 3-D printing technology, automated fiber placement technology, etc. More specifically, Khosravani et al. [4] found that the stiffness and strength of 3-D printing structural components depend on the raster angle. In detail, the results show that the highest and lowest strength have been achieved for 0° and 90° raster angles. For the AFP technology part, reasonable path planning can achieve the purpose of weight reduction and help to ensure the laying quality and efficiency. The concept of “natural path” was first proposed by Lewis [5] in 1987 and applied to the trajectory planning of automated tape-laying technology to reduce the deformation of the prepreg tape. Since then, the research on trajectory planning algorithm is mainly based on the “natural path” algorithm to improve the accuracy or efficiency and the scope of the promotion [6,7].

Generally speaking, the layers used in the laying process mainly have four angles of 0°, 90°, and ±45°, which simplify the design and analysis process under the requirement of the composite structural strength. With the development of automated placement technology, composite layers have not been limited to straight layups, and in the same layup, it has become possible to achieve constant changes in fiber angle/curvature by using advanced placement equipment.

Hyer et al. [8] first proposed the concept of replacing the straight laying path with the continuous change of fiber track curvature. Compared with the straight laying, the continuous change of fiber track curvature in the layer could significantly improve the mechanical property of the laminates with holes. Gurdal et al. [9] elaborated the concept of “variable stiffness” and proposed a new fiber curve path planning method, which made the fiber trend change linearly with the reference geometric axis, compared with the linear lamination. This method could effectively improve the buckling load of the laminates. Jegley et al. [10] studied the effect of continuous variable curvature fiber placement on the compression and shear strength of laminates, which replaced linear placement. Their results showed that the compression and shear strength of laminates with variable curvature placement could be increased by 60%. Domestically, Ma [11] studied the performance of laminates with variable curvature by using the idea of the finite element method. The modeling results showed that when the laminates were subjected to in-plane load, the buckling load of the laminates increased by about 14%. Gupta et al. [12] also used the finite element method to study the effects of fiber orientation on the mechanical properties of the natural fiber-reinforced epoxy composite. The results show that for tensile and flexural tests, all the fibers showed minimum stress at the orientation of 0° and 90°. In the Brinell hardness test, composites with a 45° angle of orientation show the best results. Fu [13] redefined the trajectory of continuous fibers by using the secondary Bezier curve method and analyzed the influence of curvature change on the buckling load of the laminate. Gu et al. [14] realized the goal of optimal layering design with the help of an ideal model and tow width model, which could obtain the optimal layering.

In the present paper, an experiment has been conducted to focus on the effect of several different path planning placements on the mechanical behavior of laminated materials. This paper is organized as follows: Section 2 describes the theoretical analysis of placement trajectory. Variable angle placement path planning is designed in Section 3, and details of experimental tests corresponding with obtained results are explained in Section 4. Finally, Section 5 outlines the conclusions.

## 2. Process Analysis of Placement Trajectory

The prepreg is usually composed of unidirectional reinforcing fiber and resin matrix, in which the resin matrix constrains the reinforcing fiber within a certain range of positions. Due to the large elastic modulus of the reinforcing fiber, the fiber is subjected to longitudinal stretching and difficult to deform, while the fiber is prone to buckling under compression. Therefore, in the planning of the laying trajectory, in addition to the shape factor of the complex curved surface, the curvature change of the prepreg should also be taken enough attention during the laying process.

**Figure 1:**
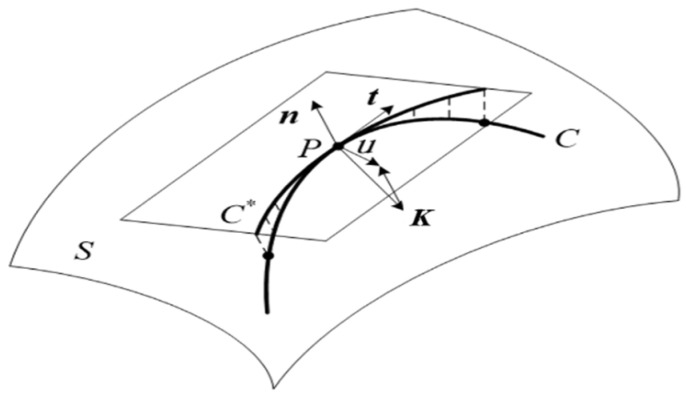
The curve curvature.

Let point P be any point of the curve ***(****C**)*** on the surface *S*, as shown in Figure 1. The curvature vector is ***K***, ***t*** is the unit tangent vector, and ***n*** is the unit normal vector of the surface S at the point P. Among them, u=n×t, so three unit vectors ***t,*** ***n***, and ***u*** are perpendicular to each other and conform to the right-hand rule. According to the theory of differential geometry, curvature vector ***K*** can be expressed in two parts: the projection on ***n*** is called normal curvature ***K****_n_*, and the projection on ***u*** is called geodesic curvature ***K_g_***. Additionally, then the curvature vector ***K*** can be written as [15] follows:

(1) $$K=K_{n}+K_{g}$$

where ***K_n_*** and ***K_g_*** represent the directions of prepreg thickness and prepreg width, respectively. ***K_n_*** only affects the bending deformation in the thickness direction. Since the prepreg is very thin (0.125–0.2 mm), compared to width, it is negligible. Therefore, the effect on the strain in the thickness direction is not significant. ***K_g_*** mainly affects the bending strain in the width direction. The geodesic curvature will mainly act on the width direction and cause deformation. Therefore, the geodesic curvature of the fiber tow must be highly valued during the laying process.

**Figure 2:**
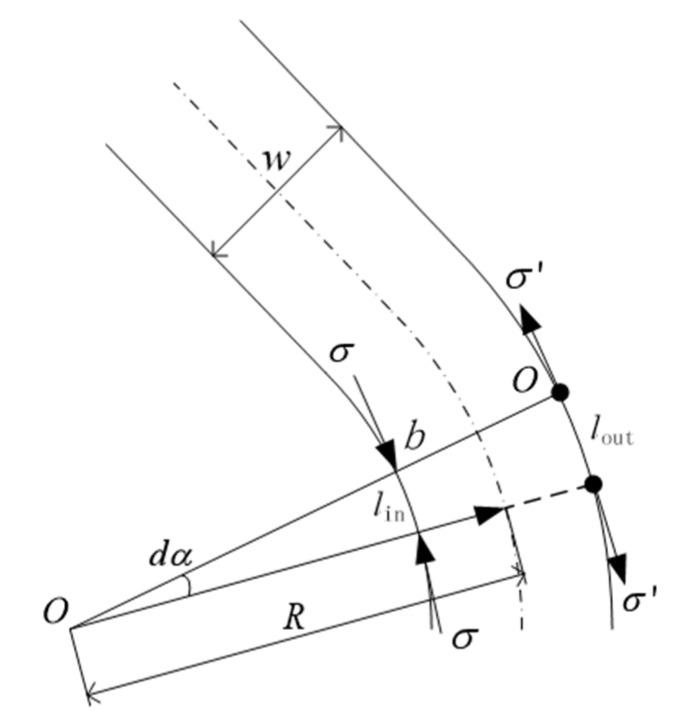
Linear placement and lateral bending of prepreg tow.

When the prepreg is laid on the plane in a straight line, the fibers at both ends of the tow have the same trajectory length; hence, there is no strain. When the tow is laterally bent due to the different length of the trajectory, some fiber of the tow will arise strain. Take a tiny area on the surface of the prepreg, as shown in Figure 2. The width of the fiber is w, in which the two sides of the selected area are perpendicular to the direction of the fiber, and the lengthening intersects point *O* with an angle of *dα*. Since the selected area is very small, the surface problem can be converted into a plane problem. If the influence of the normal curvature on the thickness direction can be neglected, the inner and outer sides are regarded as arcs, the curvature is constant, and the point *O* is the center of the circle. In the process of laying trajectory planning, the centerline of prepreg is taken as the standard, which can more directly reflect the deformation of prepreg during placement.

Here:

(2) $$R=\frac{1}{K_{g}}$$

(3) $$l_{out}=(\frac{1}{K_{g}}+\frac{w}{2})d\alpha$$

(4) $$l_{in}=(\frac{1}{K_{g}}-\frac{w}{2})d\alpha$$

In the above equations, ***K_g_*** is the geodesic curvature, *R* is the geodesic radius, ***w*** is the width of the fiber, $l_{out}$ is the distance between lengthening intersects point O and the outside boundary of the fiber, $l_{in}$ is the distance between lengthening intersects point O and the inner boundary of the fiber.

**Figure 3:**
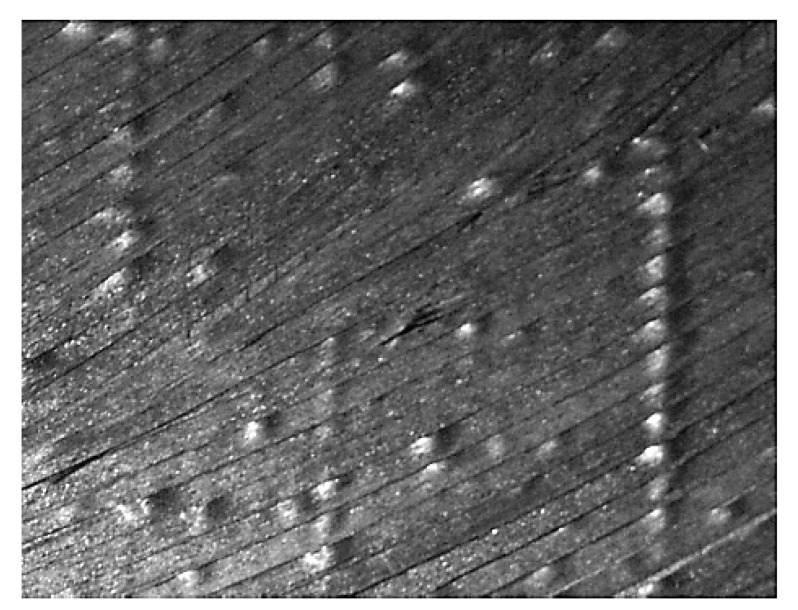
Schematic diagram of fiber buckling and wrinkling.

According to Equations (2)–(4), when the fiber has lateral bending, the path of the outer tow is longer and will be subjected to tensile stress. Since the fiber is restricted by rigidity, the tensile stress is difficult to release and only produces small deformation. The path of the inner tow is short. As a result, the inner part will be affected by the compressive stress, which will be greatly released through the fiber buckling deformation. On the macroscopic surface of the tow, wrinkled and warped may occur, as shown in Figure 3. These defects reduce the surface quality of composite components and also have a great impact on the composite’s mechanical properties. Therefore, when the geodesic curvature of the laying trajectory exceeds a certain limit, the outer fiber will bear tensile stress. Then, the tow cannot produce obvious strain, while the inner fiber bears compressive stress. The tow suffers structural instability and buckling. In this case, the compressive strain ε can be expressed as follows:

(5) $$\varepsilon =\frac{w}{\left(R+w/2\right)}=\frac{1}{\frac{1}{K_{g}w}+\frac{1}{2}}$$

It can be inferred from Equation (5) that when placing the geodesic curvature of a trajectory $K_{g}=0$, the strain of the fiber ε=0. The larger the geodesic curvature ***K_g_*** is, the larger the strain ε should be. When the strain reaches a certain limit, the tow would buckle and wrinkle, which should affect the part performance. Therefore, the geodesic curvature of the track ***K_g_*** can affect the placement process of composite materials, which is very important to judge the track laying.

## 3. Variable Angle Placement Path Planning

**Figure 4:**
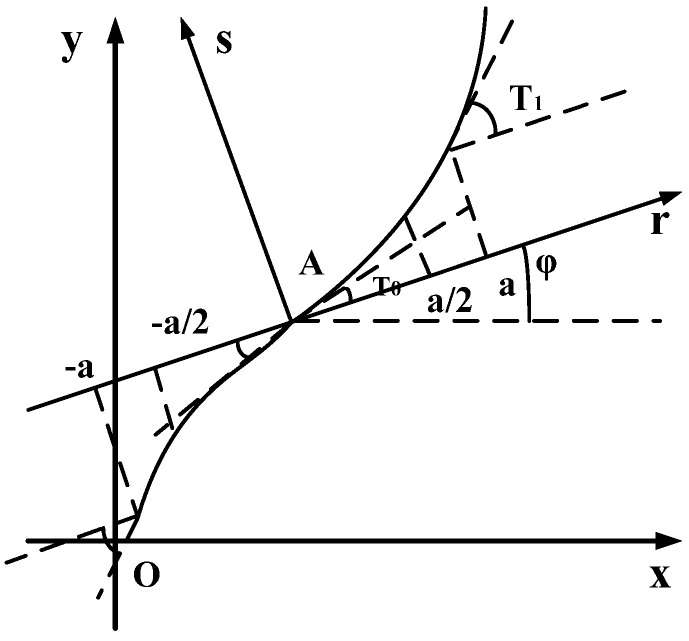
Fiber curve placement reference path planning.

The difference between a variable angle laminate and a conventional laminate is that the fiber angle of each laminate is not fixed but constantly changing on the basis of variable angle placement. Gürdal [16] further proposed a trajectory path design method, which is suitable for variable angle curve fiber placement, as shown in Figure 4. The angle θ between fiber direction and X axis is linear with the reference geometry axis, so the path of reference trajectory can be determined.

Among variable angle composite laminates, fiber curve lamination can be expressed as $\varphi (T_{0}|T_{1})$, when φ=0, $\varphi (T_{0}|T_{1})$ and $\left(T_{0}|T_{1}\right)$ are equivalent. $\varphi \pm (T_{0}|T_{1})$ indicates that there are two consecutive layers, in which $T_{0}$ and $T_{1}$ are equal in size and opposite in directions. In other words, there are two continuous layers: $\left(T_{0}|T_{1}\right)$ and $-(T_{0}|T_{1})$.

Among them, φ- the angle between the reference axis *r* and the axis *X*;

$T_{0}$—the angle between the fiber and the axis *r* at the origin of the r−s coordinate system;

$T_{1}$—the angle between the fiber and the axis *r* at the distance from the origin *a* in the r−s coordinate system.

The angle *θ* between the fiber and the axis *X* can be expressed as a function of *r*. The functional relationship between *θ* and *r* is shown in Equation (6).

(6) $$\theta (r)=\{\begin{array}{l} \frac{2}{a}(T_{1}-T_{0})+T_{0}-2(T_{0}-T_{1})\\ \frac{2}{a}(T_{0}-T_{1})r+T_{0}\\ \frac{2}{a}(T_{1}-T_{0})r+T_{0}\\ \frac{2}{a}(T_{0}-T_{1})+T_{0}-2(T_{0}-T_{1}) \end{array}\begin{array}{c} -a\leq r\leq -\frac{2}{a}\\ -\frac{2}{a}\leq r\leq 0\\ 0\leq r\leq \frac{2}{a}\\ \frac{2}{a}\leq r\leq a \end{array}$$

**Figure 5:**
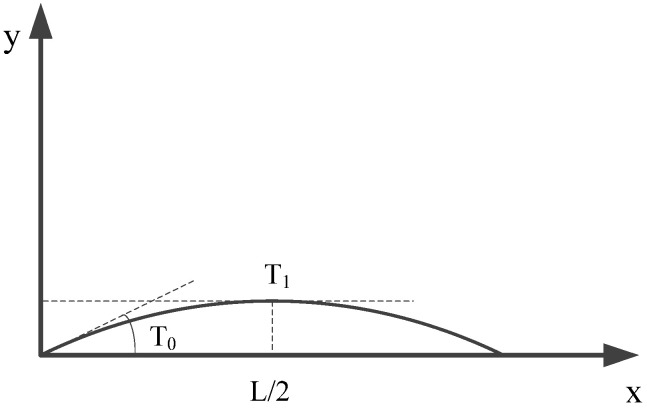
Datum path planning with different geodesic curvature.

In this paper, based on the above ideas of reference path planning, the fiber curve placement path with a geodesic curvature radius *R* of 800 mm, 1000 mm, and 1200 mm is designed independently. The planning method of the reference path is shown in Figure 5. As can be seen from the above, the fiber curve layers with a geodesic curvature radius of 800 mm, 1000 mm, and 1200 mm can be, respectively, expressed as <14|0>, <12|0>, and <10|0>. Then, according to reference [16], since θ is linear with the reference geometry axis, the fiber curve placement path can be expressed by a piecewise Equation (7).

(7) $$\theta (x)=\frac{T_{1}-T_{0}}{\frac{L}{2}}x+T_{0}=\frac{-2T_{0}}{L}x+T_{0}$$

where $T_{0}$ is the angle between the fiber curve and the axis *X* at the origin of the coordinate, and $T_{1}$ is the angle between the fiber curve and the axis *X* at the origin of the distance *L/2* coordinate, here $T_{1}=0$.

**Figure 6:**
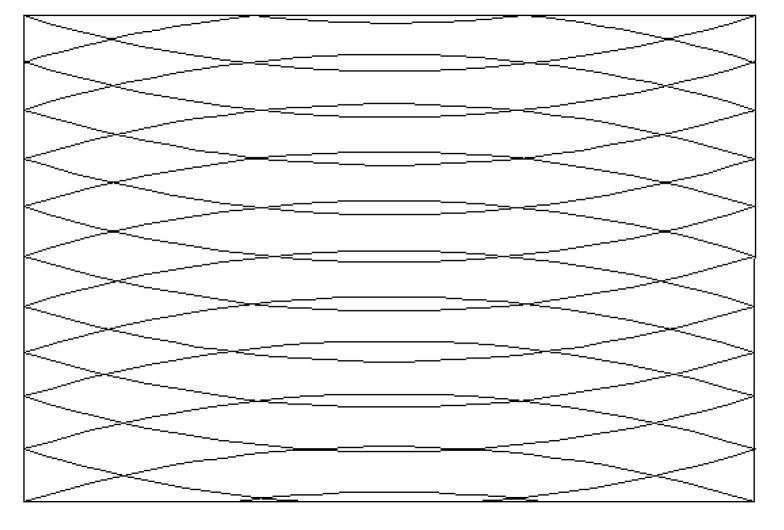
Variable angle layering.

After the reference trajectory is generated, the trajectory covers the entire plane via the translation method and forms the variable angle layer [16], which is shown in Figure 6.

## 4. Experiment

The prepreg selected for the experiment was high-strength toughened epoxy resin T300 carbon fiber prepreg UH3033-150. The prepreg tow had a slitting width of 6.35 mm, a nominal thickness of 0.156 mm, a fiber weight of 150 g/m^2^, and a resin content of 33%.

### 4.1. Preparation of Variable Angle Composite Laminates

The composite laminates were prepared by an eight-tow, seven-axis linkage laying machine. This equipment was independently developed by Nanjing University of Aeronautics and Astronautics. Each prepreg width was 6.35 mm and the size of laying composite laminate was 400mm × 300 mm. Among them, the variable angle layers of fiber curve placement were divided into three types, namely, geodesic curvature radius R = 800 mm, 1000 mm, and 1200 mm. The composite laminates with variable angles were prepared with eight layers, and the thickness of each layer was 0.156 mm, the angles of each layer were as follows:

R = 800: [<14|0>/0/0/0]_s_, R = 1000: [<12|0>/0/0/0]_s_, R = 1200: [<10|0>/0/0/0]_s_

Straight line 0°: [0]_4s_.

**Figure 7:**
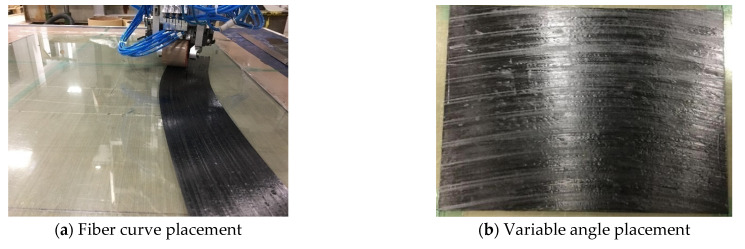
Laying process of laminate.

In combination with the path trajectory of the variable angle curve, which is shown in Figure 6, by means of the automated fiber placement machine, the prepreg with a fiber width of 6.35 mm was laid on the plane, and the fiber layers obtained different curvatures, as shown in Figure 7.

### 4.2. Vacuum Pressing and Curing

**Figure 8:**
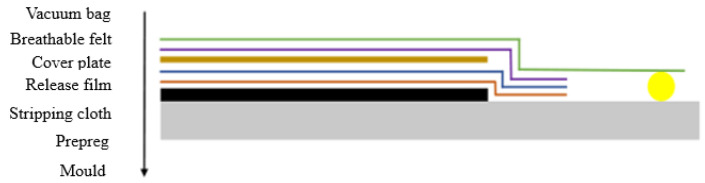
Prepressing process.

**Figure 9:**
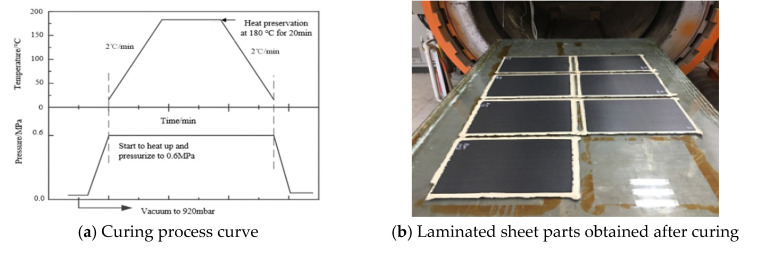
Curing process.

After the composite laminate was placed, a vacuum bag was placed on the periphery of it. Additionally, the vacuum was precompacted. The principle is shown in Figure 8. Then, the composite laminates were solidified in a hot-pressing tank. The solidification process is shown in Figure 9.

### 4.3. Specimen Preparation and Test

**Figure 10:**
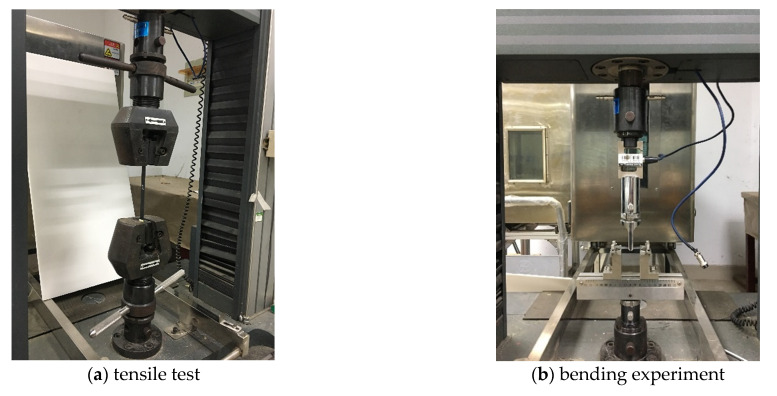
Mechanical properties test.

In this experiment, the relevant specimens were prepared with reference to the Testing Standard for Mechanical Properties of Advanced Composite Materials. The tensile test piece was a rectangular specimen of 250 mm × 15 mm × 1.25 mm, and in order to minimize the experiment error, two reinforcing pieces with the size of 56 mm × 15 mm × 1.25 mm were attached at both of the ends of the specimen. During the process, the crosshead movement speed was 2 mm/min. The tensile test process is shown in Figure 10a. The bending test piece was a rectangular specimen of 40 mm × 13 mm × 1.25 mm, which was loaded at three points and the loading speed is 1 mm/min. The bending test is shown in Figure 10b. According to the standards above, five specimens were prepared for the laminates with a straight line and different geodesic curvature curves. Additionally, the five test specimens were divided into a group for labeling and classification.

In this experiment, the equipment was the microcomputer-controlled electronic universal testing machine (model: SANS5105), which was produced by Shenzhen New SANS Material Testing Co., Ltd. During the testing process; this experiment strictly controlled the environmental temperature and humidity of 25±2 °C and 45±10%, respectively.

## 5. Experimental Results and Data Analysis

### 5.1. Summary and Data Analysis of Tensile Test

**Figure 11:**
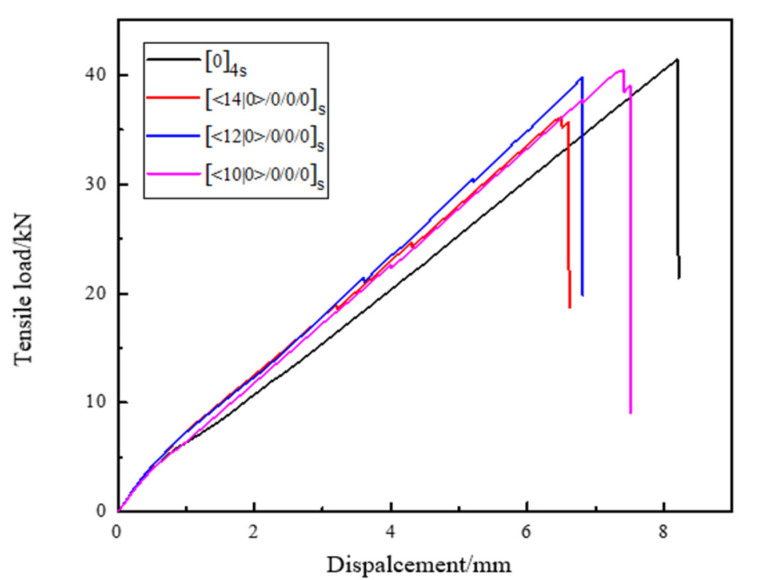
Tensile load–displacement curve of linear layup and curved layup specimens.

During the tensile test, the specimen has a small displacement under the action of the applied load. The load increases with an increase in displacement. When the applied stress reaches the breaking load of the specimen, the specimen begins to debond and occur fiber breakage. The bearing capacity is continuously decreased until the specimen instantaneously fails. The load–displacement curve of specimens with different curvatures and angles under tensile load is shown in Figure 11.

Among them according to Equation (8), the tensile strength of the specimen can be calculated as follows:

(8) $$\sigma_{\max}=\frac{F_{\max}}{bh}$$

where $\sigma_{\max}$—tensile strength, MPa; $F_{\max}$—ultimate load at failure of the specimen, *N*; b—width of the specimen, mm; h—thickness of the specimen, mm.

The calculated experimental data are collated, as shown in Table 1. Each data value is the average of the test results for five specimens.

According to Equation (8), the tensile strength of the traditional laminated plate specimens with linear lamination [0]_4s_ is 2207.84 MPa. The tensile strengths of the variable angle laminates with different geodesic curvature curves of [14|0>/0/0]_s_, [12|0>/0/0/0]_s_, [10|0>/0/0/0]_s_ are, respectively, 1927 MPa, 2121 MPa, and 2154 MPa. It can be clearly found from Table 1 that, compared with the traditional laminates with linear laminates of [0]_4s_, the tensile properties of the laminates with varying angles all decrease at different degrees. Among them, the tensile strength of [<14|0>/0/0/0]s laminates with a curvature radius of 800 mm decreases the most by 12.68%. The curvature radius of 1200 mm for [<10|0>/0/0/0]_s_ laminates decrease the least, but also by 2.392%. The reason is that the property of the carbon fiber composite is anisotropic. The tensile property along the fiber direction is good, but it will be poor when perpendicular to the fiber direction. When the specimen of composite laminates is subjected to a uniaxial tensile load, the tensile stress is dominant. However, for the traditional laminated specimens with linear laminates [0]_4s_, the fiber direction is basically consistent with the direction of the first principal stress, which is the maximum tensile stress when the force acts on the structure boundary. Therefore, the tensile properties of the fibers can be fully developed and the specimen has higher tensile strength. For layers with different geodesic curvatures of [<14|0>/0/0/0]_s_, [<12|0>/0/0/0]_s_, [<10|0>/0/0/0]_s_, under the influence of the placement angle, the fiber direction will deviate from the maximum principal stress direction to varying degrees. The fiber direction and the direction of the maximum principal stress are shown in Table 1. The greater the deviation between the direction of fiber and the direction of maximum principal stress, the more the tensile strength decreases.

### 5.2. Summary and Data Analysis of the Bending Test

**Figure 12:**
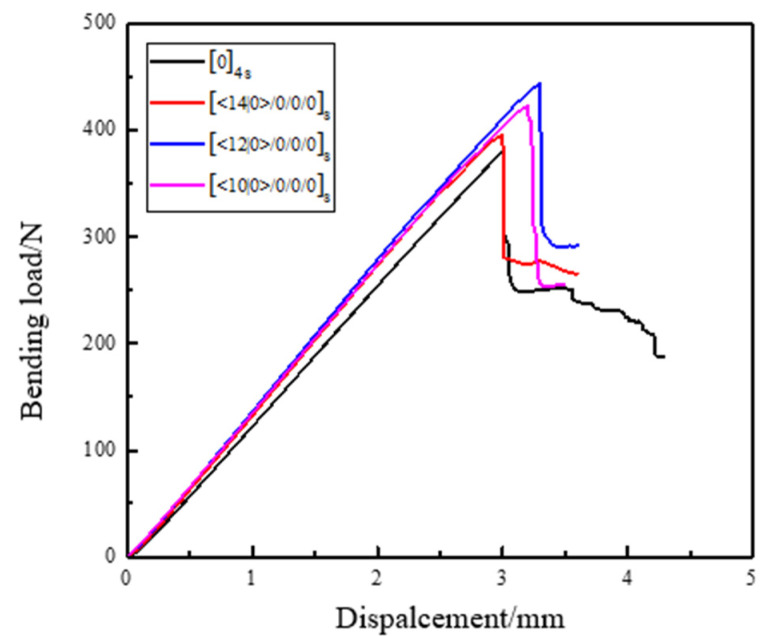
The curved load–displacement curve of a linear and curved specimen.

In the bending test, under the action of the loading indenter, the upper surface of the specimen is subjected to compressive stress, and the lower surface is subjected to tensile stress. The specimen will emit a cracking sound and a complete brittle fracture occurs when the ultimate load is reached. The load–deflection curves of specimens with different curvatures and angles under bending load are shown in Figure 12.

Among them, the bending strength of the specimen can be calculated according to Equation (9).

(9) $$\sigma_{\max}=\frac{3LF_{\max}}{2bh^{2}}$$

where $\sigma_{\max}$—bending strength, MPa; L—span between two seats; $F_{\max}$—ultimate load when the specimen is a failure, N; b—width of the specimen, mm; h—thickness of the specimen, mm.

The calculated experimental data are collated and shown in Table 2. Each data value is the average of five specimens.

According to the calculation of Equation (9), the bending strength of specimens of traditional laminates with [0]_4s_ linear laminates is 1126.42 MPa. The bending strength of the laminate specimens with different geodesic curvature curves of [14|0>/0/0]s, [12|0>/0/0/0]s, [10|0>/0/0/0]s is, respectively, 1168 MPa, 1314 MPa, and 1249M Pa. It can be clearly found from Table 2 that compared with the traditional laminates with straight-line laminates [0]4_s_, the bending strength of the laminates with varying angles of different geodesic curvature curves [<14|0>/0/0]_s_, [<12|0>/0/0/0]_s_ and [<10|0>/0/0/0]_s_ has been significantly improved. Among them, the [<12|0>/0/0/0]_s_ laminates with a radius of curvature of 1000 mm have the highest bending strength, with an increase of 16.68%. The geodesic radius of curvature is 800 mm [<14|0>/0/0/0]_s_ laminates have less increase in bending strength, but also have an increase of 3.724%. This is because, compared to the linear layup, the curve layup is longer and the span is larger in the same size. Additionally, the force distributed on the fiber per unit area is smaller with the same applied load. The fiber angle in the curve layer varies with the position so that the stress distribution inside is adjusted, and the stiffness distribution is optimized. Therefore, the failure performance is improved. In addition, since the fibers in the curved layer are curved, the fibers per unit area are longer than the linear layer. When subjected to the external bending load, the specimen fibers of the linear layer will be directly stressed, the upper layer will be subjected to compressive stress, and the lower layer will bear the tensile stress. Therefore, failure will gradually occur. For the specimen fibers with curved layers, there will be a process from bending to straightening. When they are subjected to the same bending load, they can bear more deflection than those with straight layers. Therefore, they can bear more ultimate failure load and bending strength. However, when the geodesic curvature of the fiber trajectory exceeds a certain value, the bent fibers cannot be completely retained in the prepared curved layer specimen. The fiber is truncated in the specimen, so compared with the specimen with small geodesic curvature, its performance is decreased. In conclusion, variable angle laminates with different curvatures can effectively improve their ultimate load. When the composites are subjected to bending load, the mechanical properties of composites can be effectively improved by varying the angle of curvature.

## 6. Conclusions

First, analysis of the placement process problems, which are caused by the change of prepreg curvature during the automated placement process. It is proposed that the composite placement processability can be affected by the magnitude of trajectory geodesic curvature ***K_g_***. It is very important to judge the placement ability of trajectory. Second, according to the reference trajectory of layers placement path with different curvature, the composite laminates with varying angles of [<14|0>/0/0]s, [<12|0>/0/0/0]s, [<10|0>/0/0/0]s and the composite laminates with a linear layup of [0]_4s_ are prepared. Third, for linear laminates and variable angle laminates with different curvatures, tensile and bending failure tests are, respectively, carried out. The test results show that, compared with the 0° linear layer specimen, the tensile strength of the curved laminated specimen with the curvature radius R of 1000 mm has slightly decrease by 3.889%. However, the bending strength has greatly increased by 16.68%. In the case of little difference in tensile strength, the bending strength of composite laminates can be obviously improved in the fiber curve placement. Since carbon fibers are widely used in the AFP process, here we use carbon fibers to demonstrate the effect of fiber path curvature on the mechanical properties of composites. However, for fibers such as glass fibers and Boron fibers, the result is still unclear and will be investigated in the future. Here, the fiber–matrix interface was not considered in the present study. However, it is very common in this structure due to different resin ratios, and it can greatly affect the whole strength; therefore, the relevant study will be conducted in the future. However, the result can still be useful for a different volume fraction of fibers and matrices, and the overall trend should be the same. Overall speaking, this result shows the importance of the path planning method in the AFP process and this may be used as a guideline for future AFP process.

## Figures and Tables

**Table 1:** Tensile strength of different geodesic curvature curve layers and 0° linear layer specimen.

Specimen Layup	Destructive Load (kN)	Tensile Strength (MPa)	Compared with 0° Linear Layer
[0]_4s_	41.39	2207	— —
[<14|0>/0/0/0]_s_	36.14	1927	−12.68%
[<12|0>/0/0/0]_s_	39.78	2121	−3.889%
[<10|0>/0/0/0]_s_	40.40	2154	−2.392%

**Table 2:** The bending strength of different geodesic curvature curve layers and 0° linear layer specimens.

Specimen Layup	Destructive Load (N)	Bending Strength (MPa)	Compared with 0° Linear Layer
[0]_4s_	381.3	1126	—
[<14|0>/0/0/0]_s_	395.5	1168	3.724%
[<12|0>/0/0/0]_s_	444.9	1314	16.68%
[<10|0>/0/0/0]_s_	422.9	1249	10.90%

## Data Availability

Data Sharing is not applicable.
